# Heterologous expression of genes for bioconversion of xylose to xylonic acid in *Corynebacterium glutamicum* and optimization of the bioprocess

**DOI:** 10.1186/s13568-020-01003-9

**Published:** 2020-04-15

**Authors:** M. S. Lekshmi Sundar, Aliyath Susmitha, Devi Rajan, Silvin Hannibal, Keerthi Sasikumar, Volker F. Wendisch, K. Madhavan Nampoothiri

**Affiliations:** 1 Microbial Processes and Technology Division, CSIR–National Institute for Interdisciplinary Science and Technology (NIIST), Thiruvananthapuram, 695019 Kerala India; 2 Academy of Scientific and Innovative Research (AcSIR), CSIR-National Institute for Interdisciplinary Science and Technology (CSIR-NIIST), Thiruvananthapuram, 695019 Kerala India; 3 Genetics of Prokaryotes, Faculty of Biology & CeBiTec, Bielefeld University, Bielefeld, Germany

**Keywords:** *Corynebacterium glutamicum*, Biomass, Heterologous expression, Response surface methodology (RSM), Xylose, Xylonic acid, Xylose dehydrogenase

## Abstract

In bacterial system, direct conversion of xylose to xylonic acid is mediated through NAD-dependent xylose dehydrogenase (*xylB*) and xylonolactonase (*xylC*) genes. Heterologous expression of these genes from *Caulobacter crescentus* into recombinant *Corynebacterium glutamicum* ATCC 13032 and *C. glutamicum* ATCC 31831 (with an innate pentose transporter, *araE)* resulted in an efficient bioconversion process to produce xylonic acid from xylose. Process parameters including the design of production medium was optimized using a statistical tool, Response Surface Methodology (RSM). Maximum xylonic acid of 56.32 g/L from 60 g/L xylose, i.e. about 76.67% of the maximum theoretical yield was obtained after 120 h fermentation from pure xylose with recombinant *C. glutamicum* ATCC 31831 containing the plasmid pVWEx1 *xylB*. Under the same condition, the production with recombinant *C. glutamicum* ATCC 13032 (with pVWEx1 *xylB*) was 50.66 g/L, i.e. 69% of the theoretical yield. There was no significant improvement in production with the simultaneous expression of *xylB* and *xylC* genes together indicating xylose dehydrogenase activity as one of the rate limiting factor in the bioconversion. Finally, proof of concept experiment in utilizing biomass derived pentose sugar, xylose, for xylonic acid production was also carried out and obtained 42.94 g/L xylonic acid from 60 g/L xylose. These results promise a significant value addition for the future bio refinery programs.
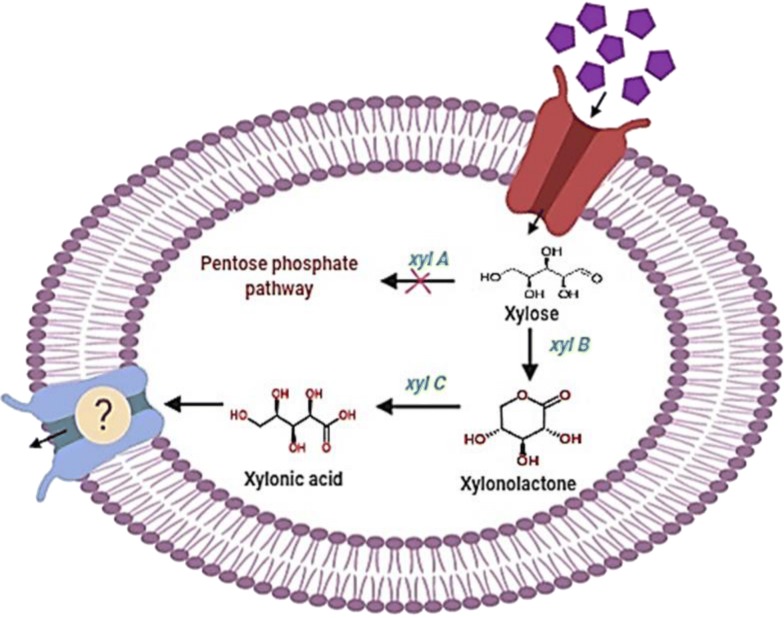

## Key points

- Made *C. glutamicum* recombinants with genes for xylose to xylonic acid conversion.
- Bioprocess development using *C. glutamicum* for xylonic acid.
- Conversion of biomass derived xylose to xylonic acid.

## Introduction

D-xylonic acid, an oxidation product of xylose, is a versatile platform chemical with multifaceted applications in the fields of food, pharmaceuticals, and agriculture. It is considered by the U.S. Department of Energy to be one of the 30 chemicals of highest value because it can be used in a variety of applications, including as a dispersant, pH regulator, chelator, antibiotic clarifying agent and health enhancer (Byong-Wa et al. 2006; Toivari et al. 2012). Xylonic acid may also be used as a precursor for bio-plastic, polymer synthesis and other chemicals such as 1,2,4-butanetriol (Niu Wei et al. 2003). Although xylonic acid production is feasible via chemical oxidation using platinum or gold catalysts, selectivity is relatively poor (Yim et al. 2017). As the pentose sugar catabolism is restricted to the majority of the industrial microbes (Wisselink et al. 2009), microbial conversion of xylose to xylonic acid gained interest. As of now, biogenic production of xylonic acid has been accomplished in various microorganisms, including *Escherichia coli*, *Saccharomyces cerevisiae* and *Kluyveromyces lactis* by introducing *xylB (*encoding *xylose dehydrogenase)* and *xylC* (encoding *xylonolactonase)* genes from *Caulobacter crescentus or Trichoderma reesei* (Nygård et al. 2011; Toivari et al. 2012; Cao et al. 2013).

As xylose is the monomeric sugar required for xylonic acid production, a lot of interest has been paid on utilizing xylose generated from lignocellulosic biomass (Lin et al. 2012). Bio-transformation of lignocellulosic biomass into platform chemicals is possible only through its conversion to monomeric sugars, mostly by pretreatment, i.e. pre-hydrolysis by alkali or acid at higher temperature or via enzymatic hydrolysis. Monomeric hexose and pentose sugars are generated from lignocellulosic biomass along with inhibitory by-products like furfural, 5-hydroxymethylfurfural, 4-hydroxybenzaldehyde that affect the performance of microbial production hosts (Matano et al. 2014). The concept of biomass refinery is getting more and more attraction for the cost effectiveness of the 2G ethanol program. Microbial production of value-added products such as biopolymers, bioethanol, butanol, organic acids and xylitol were reported utilizing the C5 stream generated by the pretreatment of biomass by different microbes like *Pichia stipitis, Clostridium acetobutylicum, Candida guilliermondii, Bacillus coagulans* (Mussatto and Teixeira 2010; Ou et al. 2011; de Arruda et al. 2011; Lin et al. 2012; Raganati et al. 2015).

Although some of the industrial strains are capable of pentose fermentation, most of them are sensitive to inhibitors of lignocellulosic biomass pretreatment. However, *Corynebacterium glutamicum* showed remarkable resistance towards these inhibitory by-products under growth-arrested conditions (Sakai et al. 2007). *C. glutamicum* is a Gram-positive, aerobic, rod-shaped, non-spore forming soil actinomycete which exhibits numerous ideal intrinsic attributes as a microbial factory to produce amino acids and high-value chemicals (Heider and Wendisch 2015; Hirasawa and Shimizu 2016; Yim et al. 2017). This bacterium has been successfully engineered towards producing a broad range of products, including diamines, amino-carboxylic acids, diacids, recombinant proteins and even industrial enzymes (Becker et al. 2018; Baritugo et al. 2018). A lot of metabolic resurrections were reported in *C. glutamicum* for the production of chemicals like amino acids, sugar acid, xylitol and biopolymers from hemicellulosic biomasses such as wheat bran, rice straw and sorghum stover (Gopinath et al. 2011; Wendisch et al. 2016; Dhar et al. 2016).

Since *C. glutamicum* lacks the genes for the metabolic conversion of xylose to xylonic acid, the heterologous expression of xylose dehydrogenase (*xylB*) and xylonolactonase (*xylC*) genes from *Caulobacter crescentus* was attempted. In addition to ATCC 13032 wild type, we also explored the *C.glutamicum* ATCC 31831 culture which contains a pentose transporter gene (*araE*) which enables the uptake of pentose sugar (Kawaguchi et al. 2009; Choi et al. 2019). Both *xylB* and *xylC* genes individually, as well as together as *xylBC*, were amplified from xylose operon of *C. crescentus* and the plasmids were transformed to both *C. glutamicum* strains and checked the xylonic acid production.

## Materials and methods

### Microbial strains and culture conditions

Microbial strains and plasmids used in this study are listed in Table 1. For genetic manipulations, *E. coli* strains were grown at 37 °C in Luria–Bertani (LB) medium. *C. glutamicum* strains were grown at 30 °C in Brain Heart Infusion (BHI) medium. Where appropriate, media were supplemented with antibiotics. The final antibiotic concentrations for *E. coli* and *C. glutamicum* were 25 μg/ml of kanamycin. Culture growth was measured spectrophotometrically at 600 nm using a UV–VIS spectrophotometer (UVA-6150, Shimadzu, Japan).

**Table 1:** Microbial strains, plasmids and primers used in the study

Strains and vectors	Descriptions	References
Microbial strains		
*Corynebacterium glutamicum*	ATCC13032, wild type (WT)	Abe et al. (1967)
*Corynebacterium glutamicum*	ATCC 31831	Kinoshita et al. (2004)
*Escherichia coli* DH5α	*Fthi*-*1 endA1 hsdr17*(*r*-*, m*-) *supE44 _lacU169 f80lacZ_M15) recA1 gyrA96 relA1*	Hanahan and Harbor (1983)
Plasmid vectors		
*pVWEx1*	Kan^r^; *E. coli*-*C. glutamicum* shuttle vector	Peters-Wendisch et al. (2001)
*pEKEx3 xylXABCD*	Spec^r^; pEKEx3 derivative for the regulated expression of *xylXABCD*_Cc_ of *C. crescentus*	This study
Primers (sequences 5′–3′)		
xylB-pVW-fw	CGCCAAGCTTGCATGC**CTGCAG**TAAAGGAGATATACATATGTCCTCAGCCATCTATCC	This study
xylB-pVW-rw	CGAGCTCGGTACCCGG**GGATCC**CTTCACGCTGGGCCGGGATG	This study
xylC-pVW-fw	CGCCAAGCTTGCATGC**CTGCAG**TAAAGGAGATATACATATGACCGCTCAAGTCACTTG	This study
xylC-pVW-rw	CGAGCTCGGTACCCGG**GGATCC**GGGCGTGCGGTTAGACAAGG	This study
xylBC-pVW-fw	TGTTTAAGTTTAGTGGATGGGATGACCGCTCAAGTCACTTGCGTATGGG	This study
xylBC-pVW-fw	CCCATCCACTAAACTTAAACATCAACGCCAGCCGGCGTCGATCC	This study

### Molecular techniques and strain construction

Standard molecular techniques were done according to the protocol described by (Sambrook et al. 2006). Genomic DNA isolation was done with Gen Elute genomic DNA isolation kit (Sigma, India). Plasmid isolation was done using Qiagen plasmid midi kit (Qiagen, Germany). Polymerase chain reaction (PCR) was performed using automated PCR System (My Cycler, Eppendorff, Germany) in a total volume of 50 μl with 50 ng of DNA, 0.2 mM dNTP in PrimeSTAR™ buffer (Takara), and 1.25 U of PrimeSTAR™ HS DNA polymerase (Takara) and the PCR product was purified by QIA quick PCR purification kit (Qiagen, Germany) as per the instructions provided by the manufacturers. Competent *E. coli* DH5α cells were prepared by Transformation and Storage Solution (TSS) method and transformed by heat shock (Chung and Miller 1993). The *C. glutamicum* competent cells were electroporated to achieve the transformation (van der Rest et al. 1999).

Xylose dehydrogenase (*xylB*) and xylonolactonase (*xylC*) and *xylBC* genes together of *Caulobacter crescentus* were amplified from the xylose-inducible *xylXABCD* operon (CC0823–CC0819) (Stephens et al. 2007) by polymerase chain reaction (PCR) with appropriate primers as shown in Table 1 and the purified PCR products (747 bp *xylB*, 870 bp *xylC* and 1811 bp *xylBC*) were verified by sequencing and cloned into the restriction digestion site (*Bam* HI/*Pst* I) of pVWEx1 shuttle vector. The engineered plasmids so-called pVWEx1*xylB*, pVWEx1*xylC* and pVWEx1*xylBC* were transformed into *E. coli* DH5α and the transformants bearing pVWEx1 derivative were screened in LB medium supplemented with kanamycin (25 µg mL^−1^). Competent cells of *C. glutamicum* ATCC 13032 and ATCC 31831 were prepared and the plasmids were electroporated into both the *C. glutamicum* strains with parameters set at 25 μF, 600 Ω and 2.5 kV, yielding a pulse duration of 10 ms and the positive clones were selected in LBHIS kanamycin (25 µg mL^−1^) plates (van der Rest et al. 1999).

### Fermentative production of xylonic acid by *C. glutamicum* transformants

For xylonic acid production, *C. glutamicum* was inoculated in 10 ml of liquid medium (BHI broth) in a test tube and grown overnight at 30 °C under aerobic condition with shaking at 200 rpm. An aliquot of the 10 ml culture was used to inoculate 100 ml CGXII production medium (Keilhauer et al. 1993) containing 35 g/L xylose and 5 g/L glucose as carbon sources, kanamycin (25 µg mL^−1^). IPTG (1 mM) induction was done along with the inoculation. Fermentation was carried out in 250 mL Erlenmeyer flasks containing 100 mL production medium and incubated as described above. Samples were withdrawn at regular intervals to determine sugar consumption and xylonic acid production. Since *xylB* transformant was found to be the best producer, a comparison of it with *C. glutamicum* ATCC 13032 having *xylB* gene was also carried out to see whether the inbuilt *araE* pentose transporter in ATCC 31831 has any advantage over wild type ATCC 13032.

### Media engineering by response surface methodology (RSM)

Response surface methodology was applied to identify the operating variables that have a significant effect on xylonic acid production. A Box Behnken experimental design (BBD) (Box and Behnken 1960) with four independent variables (selected based on single parameter study, data not shown) that may affect xylonic acid production, including (NH_4_)_2_SO_4_ (2.5–12.5 g/L), urea (4.5–18.5 g/L), xylose (30–90 g/L) and inoculum (7.5–1.125%) were studied at three levels − 1, 0 and + 1 which correspond to low, medium and high values respectively. Responses were measured as titer (g/L) of xylonic acid. The statistical as well as numerical analysis of the model was evaluated by analysis of variance (ANOVA) which included p-value, regression coefficient, effect values and F value using Minitab 17 software. Studies were performed using *C. glutamicum* ATCC 31831 harboring pVWEx1-*xylB*.

### Dilute acid pretreatment of the biomass

The rice straw was crushed into fine particle (size of 10 mm) and pre-soaked in dilute acid (H_2_SO_4_) for 30 min, pretreated with 15% (w/w) biomass loading and 1% (w/w) acid concentration at 121 °C for 1 h. After cooling, the mixture was neutralized to pH 6–7 using 10 N NaOH. The liquid portion, i.e. acid pretreated liquor (APL) rich in pentose sugar (xylose) was separated from the pretreated slurry and lyophilized to concentrate to get desired xylose level which was estimated prior to the shake flask fermentation studies.

### Quantification of sugars and xylonic acid in fermentation broth

The qualitative and quantitative analysis of sugars and sugar acid (xylonic acid) was performed using an automated high-performance liquid chromatography (HPLC) system (Prominence UFLC, Shimadzu, Japan) equipped with auto-sampler, column oven and RI Detector. The monomeric sugars (xylose and glucose) were resolved with Phenomenex Rezex RPM Pb^+^ cation exchange monosaccharide column (300 × 7.5 mm) operated at 80 °C. MilliQ water (Millipore) with a flow rate of 0.6 mL/min was used as the mobile phase. For xylonic acid detection, Phenomenex organic acid column (250 mm × 4.6 mm × 5 µm) operated at 55 °C was used with a mobile phase of 0.01 N H_2_SO_4_ at a flow rate of 0.6 mL/min. The samples were centrifuged (13,000 rpm for 10 min at 4 °C) and filtered using 0.2 µm filters (Pall Corporation, Port Washington, New York) for analysis.

## Results

### Xylose utilization and xylonic acid production by *C. glutamicum* transformants

**Fig. 1:**
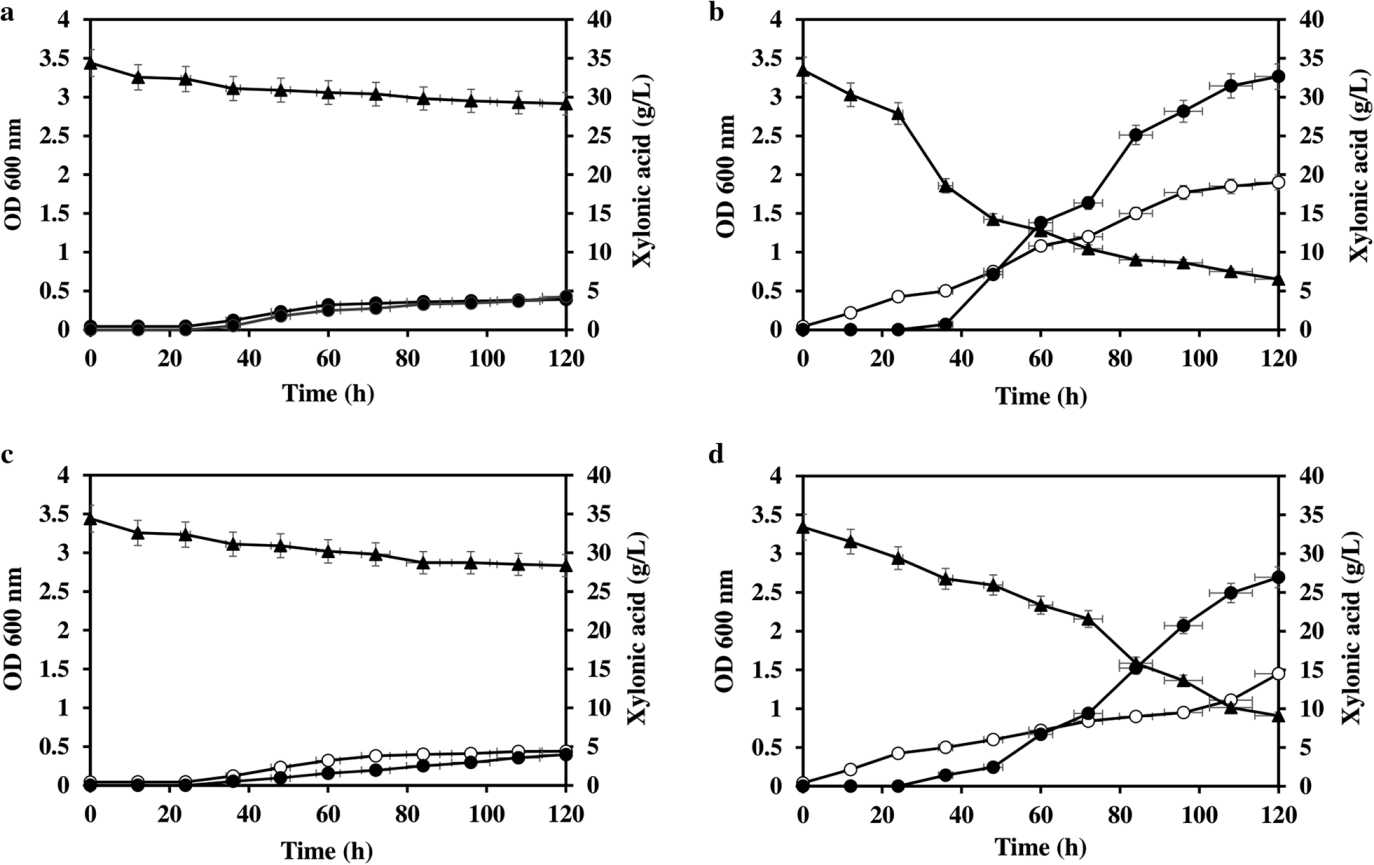
Xylose consumption (35 g/L) (*closed triangle*), xylonic acid production (closed circle) and growth curve (open circle) of *C. glutamicum* ATCC 31831 (**a**) pVWEx1 (**b**) pVWEx1-*xyl B* (**c**) pVWEx1-*xylC* (**d**) pVWEx1-*xyl BC* respectively

*Corynebacterium glutamicum* recombinants expressing *xylB*, *xylC* and *xylBC* were constructed. The xylose dehydrogenase and xylonolactonase genes were cloned into IPTG-inducible expression vector pVWEx1 and transformed into *C. glutamicum* ATCC 31831. To check xylonic acid production from xylose, the *C*. *glutamicum* ATCC 31831 transformants harboring pVWEx1-*xylB,* pVWEx1-*xylC* and pVWEx1-*xylBC* were cultivated in CGXII medium containing 5 g/L of glucose as the carbon source for initial cell growth and 35 g/L of xylose as the substrate for xylonic acid production. Cell growth, xylose consumption and xylonic acid production were analyzed during the incubation for a desired period of interval. From analysis, it is clear that compared to the control strain with empty vector (Fig. 1a), the transformants harboring pVWEx1-*xylB* picked up growth very fast compared to the other transformants and utilized xylose effectively (77.2% utilization after 120 h) and resulted in maximum production of 32.5 g/L xylonic acid (Fig. 1b). The pVWEx1-*xylB*C harboring strain produced 26 g/L xylonic acid (Fig. 1d), whereas pVWEx1-*xylC* showed neither any significant xylose uptake nor xylonic acid production (Fig. 1c).

### Box–Behnken experimental design (BBD) and operational parameter optimization

The objective of the experimental design was medium engineering for maximum xylonic acid production. There were a total of 15 runs for optimizing the four individual parameters in the current BBD. Experimental design and xylonic acid yield are presented in Table 2. The polynomial equation obtained for the model was as below:

$$\begin{aligned} {\text{Xylonic acid }}\left( {{{\text{g}} \mathord{\left/ {\vphantom {{\text{g}} {\text{L}}}} \right. \kern-0pt} {\text{L}}}} \right)\; = \; & - 4 8. 7 { }{-} \, 0. 4 5 {\text{ X}}_{ 1} + { 3}. 5 {\text{ X}}_{ 2} + 0. 2 20{\text{ X}}_{ 3} + { 2}.0 5 8 {\text{ X}}_{ 4} \\ & {-} \, 0.0 1 9 {\text{ X}}_{ 1}^{ 2} {-} \, 0. 2 1 3 9 {\text{ X}}_{ 2}^{ 2} {-} \, 0.0 4 2 3 {\text{ X}}_{ 3}^{ 2} {-} \, 0.0 1 9 4 3 {\text{ X}}_{ 4}^{ 2} \\ & {-} \, 0.0 7 5 {\text{ X}}_{ 1} {\text{X}}_{ 2} + \, 0.0 4 1 6 {\text{ X}}_{ 1} {\text{X}}_{ 3} {-} \, 0.0 1 1 9 {\text{ X}}_{ 1} {\text{X}}_{ 4} \\ & + \, 0. 5 2 6 {\text{ X}}_{ 2} {\text{X}}_{ 3} + \, 0.0 4 8 2 {\text{ X}}_{ 2} {\text{X}}_{ 4} {-} \, 0.00 1 2 8 {\text{ X}}_{ 3} {\text{X}}_{ 4} \\ \end{aligned}$$

where X_1_, X_2_, X_3_ and X_4_ are xylose, (NH4)_2_SO_4_, urea and inoculum concentration respectively. Maximum production efficiency (0.47 g^−1^ L^−1^ h^−1^) was observed with Run No.13 where the concentration of parameters was urea 11.5 g/L, xylose 60 g/L, (NH_4_)_2_SO_4_ 7.5 g/L and inoculum 1.125% and xylonic acid titer was 56.32 g/L. It indicates that (NH_4_)_2_SO_4_, inoculum concentration and xylose have a significant positive effect than urea on xylonic acid yield.

**Table 2:** Box–Behnken experimental design matrix with experimental values of xylonic acid production by *Corynebacterium glutamicum* ATCC 31831

Run order	Urea (g/L)	Xylose (g/L)	(NH_4_)_2_SO_4_ (g/L)	Inoculum (% v/v)	Xylonic acid (g/L)
1	11.5	60	7.5	11.25	56.119
2	11.5	90	2.5	11.25	59.792
3	11.5	30	12.5	7.5	25.061
4	4.5	30	7.5	15	21.359
5	18.5	60	2.5	15	52.481
6	11.5	30	2.5	7.5	25.061
7	11.5	90	12.5	15	58.418
8	4.5	60	12.5	11.25	30.341
9	18.5	90	7.5	15	58.795
10	4.5	90	7.5	11.25	45.749
11	18.5	60	12.5	15	48.982
12	11.5	60	7.5	15	56.018
13	11.5	60	7.5	15	*56.318*
14	18.5	30	7.5	11.25	28.349
15	4.5	60	2.5	7.5	28.816

Maximum conversion of xylose to xylonic acid indicated in italic

**Fig. 2:**
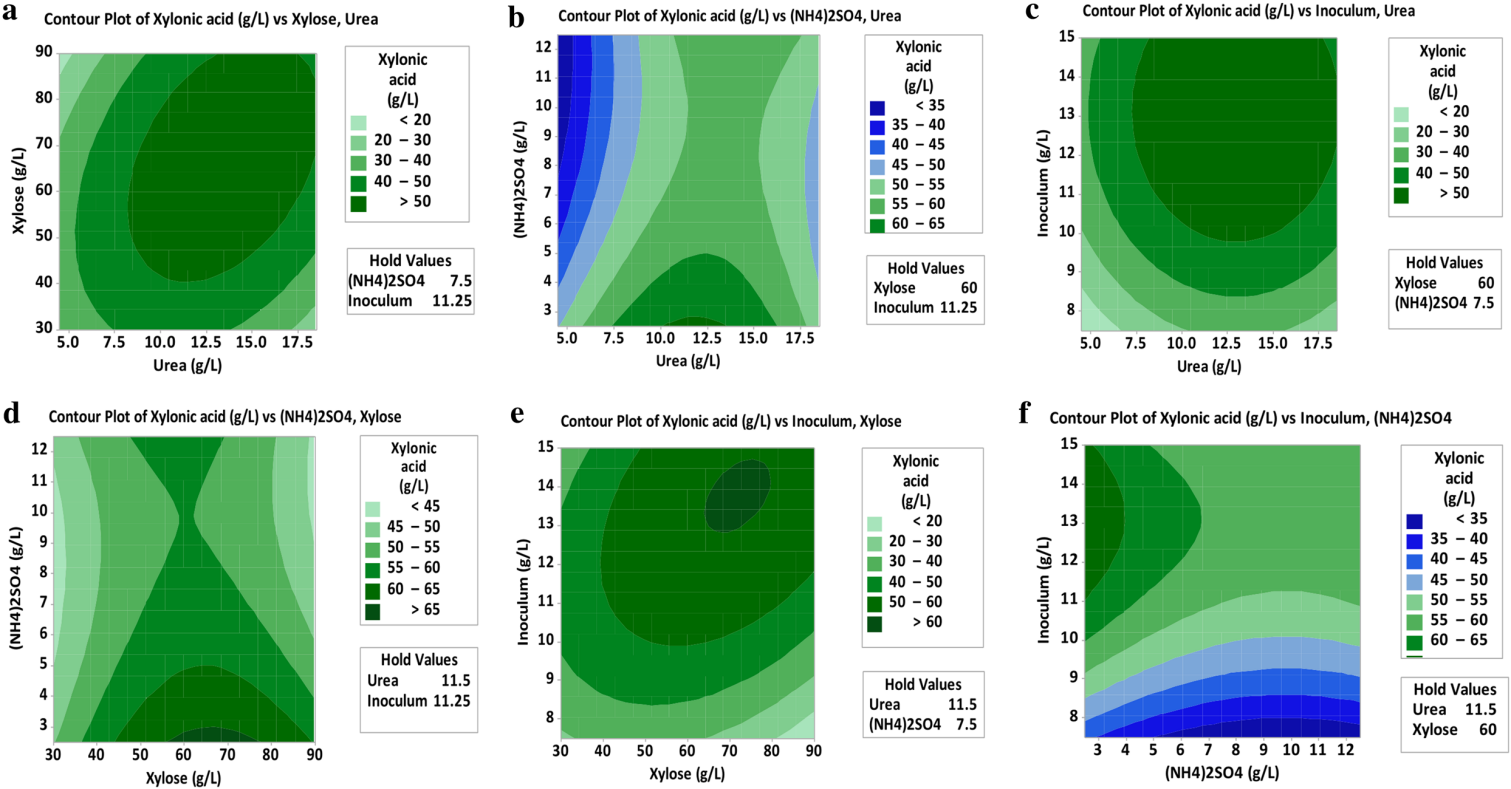
Response surface methodology-contour plots showing the effect of various parameters on xylonic acid production by *C.glutamicum* ATCC 31831. **a** Effect of inoculum and xylose. **b** Effect of xylose and urea. **c** Effect of (NH_4_)_2_SO_4_ and urea. **d** Effect of inoculum and (NH_4_)_2_SO_4_. **e** Effect of (NH_4_)_2_SO_4_ and xylose **f** Effect of inoculum and urea

Response surface curves were plotted to find out the interaction of variables and to determine the optimum level of each variable for maximum response. The contour plot showing the interaction between a pair of factors on xylonic acid yield is given in Fig. 2a–f. Major interactions studied are of inoculum and xylose concentration (a), xylose and urea concentration (b), (NH_4_)_2_SO_4_ and urea concentration (c), effect of inoculum and (NH_4_)_2_SO_4_ concentration (d), effect of (NH_4_)_2_SO_4_ and xylose concentration (e) and the interaction of inoculum and urea concentration (f).

The ANOVA of response for xylonic acid is shown in Table 3. The R^2^ value explains the variability in the xylonic acid yield associated with the experimental factors to the extent of 97.48%.

**Table 3:** Analysis of variance for xylonic acid production using *C. glutamicum* ATCC 31831

Source	DF	Adj SS	Adj MS	F	P
Regression	12	3583.09	298.591	6.45	0.142
Linear	4	1688.34	422.234	9.11	0.101
Square	4	1249.59	312.398	6.74	0.133
Interaction	4	284.83	71.208	1.54	0.431
Residual error	2	92.66	46.328		
Lack-of-fit	1	0.00	92.657		
Pure error	1		0.000		
Total	14	3675.75			

S = 6.80649, R-Sq = 97.48%, R-Sq (pred) = 0.00% and R-Sq (adj) = 82.35%

### Role of *araE* pentose transporter for enhanced uptake of xylose and xylonic acid production

**Fig. 3:**
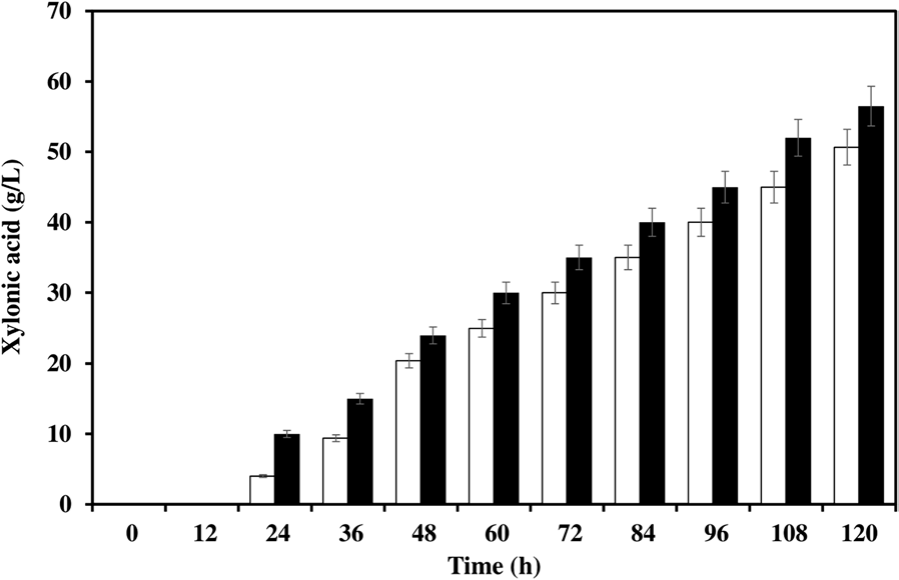
Xylonic acid production by *C. glutamicum* ATCC 13032 (open bar) and *C. glutamicum* ATCC 31831 (closed bar) harbouring plasmid pVWEx1-*xylB*

Using the designed medium standardized for *C. glutamicum* ATCC 31831, which possesses an arabinose and xylose transporter encoded by *araE*, a comparative production study was carried out with recombinant *C. glutamicum* ATCC 13032. Both the strains grew well in the CGXII production medium and metabolized xylose to xylonic acid. After 120 h fermentation, the recombinant strain, ATCC 13032 produced 50.66 g/L of xylonic acid whereas ATCC 31831 produced 56.32 g/L (Fig. 3). It was observed that better uptake of the pentose sugar was also exhibited by *C. glutamicum* ATCC 31831, i.e., 75% consumption compared to 60% by ATCC 13032 after 120 h fermentation and same the case with culture growth where ATCC 31831 showed better growth (10× dilution of culture broth for spectrophotometric reading (Additional file 1: Figure S1).

### Xylonic acid from rice straw hydrolysate

**Fig. 4:**
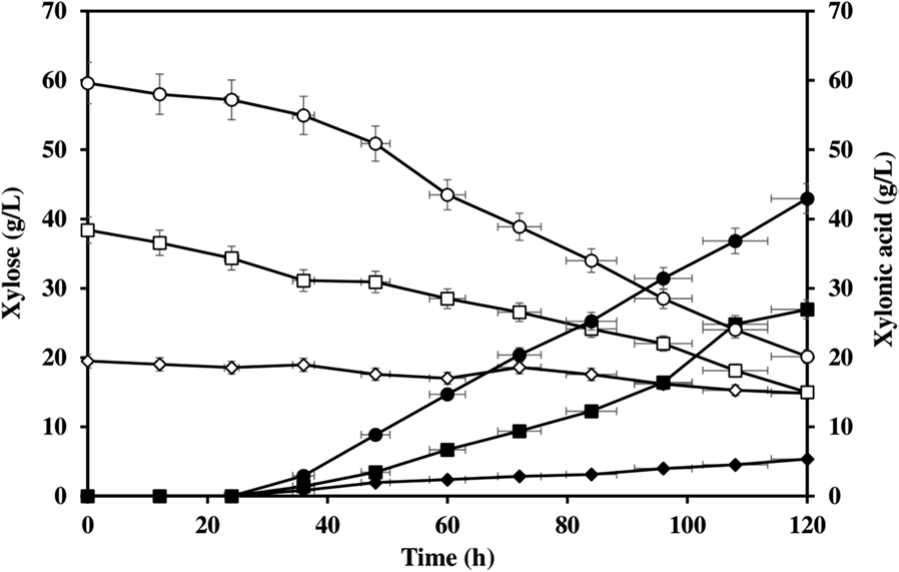
Xylose utilization (open symbols) and xylonic acid production (closed symbols) by *C. glutamicum* ATCC 31831 (pVWEx1-*xylB*) in rice straw hydrolysate containing different concentrations of xylose 20 g/L (open diamond), 40 g/L (open square) and 60 g/L (open circle). Xylonic acid production from 20 g/L xylose (closed diamond), 40 g/L xylose (closed square) and 60 g/L xylose (closed circle)

Fermentation was carried out in rice straw hydrolysate using *C. glutamicum* ATCC 31831 (pVWEx1-*xylB*). The strain could grow in different xylose concentrations (of 20, 40, and 60 g/L) in rice straw hydrolysate, and after 120 h fermentation, maximum titer obtained was 42.94 g/L xylonic acid from 60 g/L xylose (Fig. 4). A production yield of 58.48% xylonic acid in hydrolysate is remarkable for sugar acid production with engineered strain of *C. glutamicum* which is quite tolerant to the inhibitors present in the hydrolysate.

## Discussion

Heterologous expression of genes for the production of varied value-added chemicals were successfully carried out in *C. glutamicum*, for example, the production of amino acids, sugar alcohol, organic acid, diamines, glycolate and 1,5-diaminopentane (Buschke et al. 2013; Meiswinkel et al. 2013; Zahoor et al. 2014; Pérez-García et al. 2016; Dhar et al. 2016). *C. glutamicum* being a versatile industrial microbe and the availability of genetic engineering tools makes it a rapid and rational manipulation host for diverse platform chemicals. Most corynebacteria are known not to utilize xylose as carbon source. The absence of xylose metabolizing genes restricts the growth of *Corynebacterium* in pentose rich medium. To develop an efficient bioconversion system for xylonic acid synthesis, the genes of *Caulobacter crescentus* were expressed in *C. glutamicum.* The resulting transformants *C. glu*-pVWEx1-*xylB* and *C.glu*-pVWEx1-*xylBC* were able to grow in mineral medium containing xylose and converted it into corresponding pentonic acid.

Xylose can be metabolized in four different routes (I) The oxido-reductase pathway, (II) The isomerase pathway, (III) The Weimberg pathway, an oxidative pathway and (IV) The Dahms pathway (Cabulong et al. 2018). Xylose once inside the cell gets converted to xylonolactone and then into xylonic acid on the expression of two genes namely, *xylB* (xylose dehydrogenase) and *xylC* (xylonolactonase). These two enzymes are involved in both the Weimberg and Dahms pathway where xylose is metabolized to xylonic acid (Brüsseler et al. 2019). In the present study, it is observed that only the xylose dehydrogenase enzyme activity is good enough for xylonic acid production. Without the dehydrogenase activity, the lactonase activity alone cannot do the conversion of xylose to xylonic acid. Further, the xylonolactonase expression along with xylose dehydrogenase resulted in xylonic acid production but not that efficient as dehydrogenase alone with the case of *C. glutamicum*. It is reported that, xylonolactone once formed can be converted to xylonic acid either by the spontaneous oxidation of lactone or through the enzymatic hydrolysis of xylonolactonase enzyme (Buchert and Viikari 1988). *Corynebacterium glutamicum* being an aerobic organism, direct oxidation of xylonolactone to xylonic acid is more favorable inside the cell. Previous studies have also shown that xylose dehydrogenase (*xylB*) activity alone can result in the production of xylonic acid (Yim et al. 2017).

*Corynebacterium glutamicum* ATCC 31831 grew on pentose as the sole carbon source. The gene cluster responsible for pentose utilization comprised a six-cistron transcriptional unit with a total length of 7.8 kb. The sequence of the *C. glutamicum* ATCC 31831 *ara* gene cluster containing gene *araE*, encodes pentose transporter, facilitates the efficient uptake of pentose sugar (Kawaguchi et al. 2009). Previous studies have also reported the role of *araE* pentose transporter in *Corynebacterium glutamicum* ATCC 31831 and its exploitation for the production of commodity chemicals like 3HP and ethanol (Becker et al. 2018). In the present study, *Corynebacterium glutamicum* ATCC 31831 with an inbuilt *araE* pentose transporter exhibited effectual consumption of xylose as well as its conversion to xylonic acid. Further studies have to be done to explore the role of the same *araE* pentose transporter as an exporter for xylonic acid.

*Micrococcus* spp., *Pseudomonas*, *Kluveromyces lactis, Caulobacter*, *Enterobacter*, *Gluconobacter*, *Klebsiella* and *Pseudoduganella danionis* (ISHIZAKI et al. 1973; Buchert et al. 1988; Buchert and Viikari 1988; Toivari et al. 2011; Wiebe et al. 2015; Wang et al. 2016; Sundar Lekshmi et al. 2019) are the non-recombinant strains reported for xylonic acid production. Among which *Gluconobacter oxydans* is the prominent wild-type strain exhibits higher titers of xylonic acid up to 100 g L^−1^ (Toivari et al. 2012). Although these strains are capable of producing xylonic acid from pure sugar, they fail to perform as an industrial strain since some are opportunistic pathogen grade and they are not tested in hydrolysate medium may be due to their lower tolerance towards lignocellulosic inhibitors. There was an earlier report on recombinant *C. glutamicum* ATCC 13032 produced 6.23 g L^−1^ of xylonic acid from 20 g L^−1^ of xylan (Yim et al. 2017). In this study they have employed multiple modules, (i) xylan degradation module (ii) conversion module from xylose to xylonic acid by expression of *xdh* gene and (iii) xylose transport module by expression of *xylE* gene, and optimized gene expression introducing promoters (Yim et al. 2017). The product titers with *C. glutamicum* ATCC 31831 presented in this study are comparable with other wild type and recombinant strains (Table 4) and the volumetric productivity in the feed phase can outperform the titers published employing the recombinant *C. glutamicum* ATCC 13032.

**Table 4:** Comparison of xylonic acid production and productivity by the best xylonic acid producers

Microorganism	d-xylose (g/l)	d-xylonate (g/l)	Yields (g/g)	Volumetric productivity (g/l/h)	Specific productivity [g(g/biomass)/h]	PH	Biomass (g/l)	Process	References
*Gluconobacter oxydans* (ATCC 621)	100	109	1.1	2.5	~ 1.5	5.5	1.7	Batch	Buchert et al. (1988)
*Gluconobacter oxydans* (ATCC 621)	100	107	1.1	2.2	~ 1.5	4.5	1.3	Batch	Buchert et al. (1988)
*Pseudomonas fragi* (ATCC 4973)	150	162	1.1	1.4	0.2	6.5	6.9	Batch	Buchert et al. (1988)
*Pseudomonas putida*	~ 0.4	~ 0.4	~ 1	~ 1.9	~ 0.7	6.8	2.9	Continuous	Meijnen et al. (2009)
*Enterobacter cloacea*	200	190	~ 1	~ 1.6	–	6.5	nd	Batch	Ishizaki et al. (1973)
*Escherichia coli*	40	39	1.0	1.1	0.14	7.0	~ 8	Batch	Liu et al. (2012)
*Saccharomyces cerevisiae Xyd 1*	20	4	0.4	0.03	0.007	5.5	4.6	Batch	Toivari et al. (2010)
*Saccharomyces cerevisiae* SUS2DD	23	3	0.4	0.02	0.006	5.5	5.3	Batch	Toivari et al. (2012)
*Saccharomyces cerevisiae xylB*	23	17	0.8	0.23	0.06	5.5	5	Batch	Toivari et al. (2012)
*Kluyveromyces lactis Xyd 1*	23	8	0.4	0.13	0.01	5.5	9	Batch	Nygård et al. (2011)
*Corynebacterium glutamicum* (ATCC 13032)	20	6.23	1.04	1.02	–	–	–	Batch	Yim et al. (2017)
*Corynebacteriumglutamicum* (ATCC 31831)	60	56.32	~ 1	0.93	–	5.5	1.4	Batch	This study

Media engineering was carried out with the statistical tool response surface methodology (RSM) for the enhanced production of xylonic acid. The Box–Behnken model with experimental values containing 15 runs was designed for the optimization study. RSM aided to narrow down the most influencing parameters and its optimization on xylonic acid production. The engineered strain produced up to 56.3 g/L of xylonic acid and is characterized by high volumetric productivity and maximum product yield of 76.67% under optimized conditions applying defined xylose/glucose mixtures in synthetic medium. One of the major challenges is the range of acidic and furan aldehyde compounds released from lignocellulosic pre-treatment. Here, the recombinant *C. glutamicum* ATCC 31831 could resist the inhibitors present in rice straw hydrolysate and produced xylonic acid nearly to 58.5% of the maximum possible yield.

The challenges involve getting sufficient xylose after pretreatment and also the separation of xylonic acid from the fermented broth. For the industrial application, downstream processing of xylonic acid is very important. Ethanol precipitation and product recovery by extraction are the two interesting options described for the purification of xylonic acid from the fermentation broth (Liu et al. 2012). With this industrially streamlined recombinant strain a highly profitable bioprocess to produce xylonic acid from lignocellulosic biomass as a cost-efficient second-generation substrate is well within the reach. The one-step conversion of xylose to xylonic acid and the bioprocess developed in the present study favors pentose sugar utilization in rice straw in a straight forward and cost-effective method. The proof of concept showed the simultaneous utilization of biomass-derived sugars (C5 and C6) and it has to be investigated in detail.

## Supplementary information

**Additional file 1: Figure S1.** Growth (circles) and xylose consumption (triangles) by *C. glutamicum* ATCC 13032 (pVWEx1-*xylB*) (open symbols) and *C. glutamicum* ATCC 31831 (pVWEx1-*xylB*) (closed symbols) in CGXII medium containing 60 g/L xylose.

## Data Availability

All data generated or analysed during this study are included in this published article and its additional files.
