# Supplementary figures and images for: Heterologous expression of genes for bioconversion of xylose to xylonic acid in Corynebacterium glutamicum and optimization of the bioprocess
Source: AMB Express. 2020 Apr 15;10:68. doi: 10.1186/s13568-020-01003-9 (PMC7158973; doi:10.1186/s13568-020-01003-9)

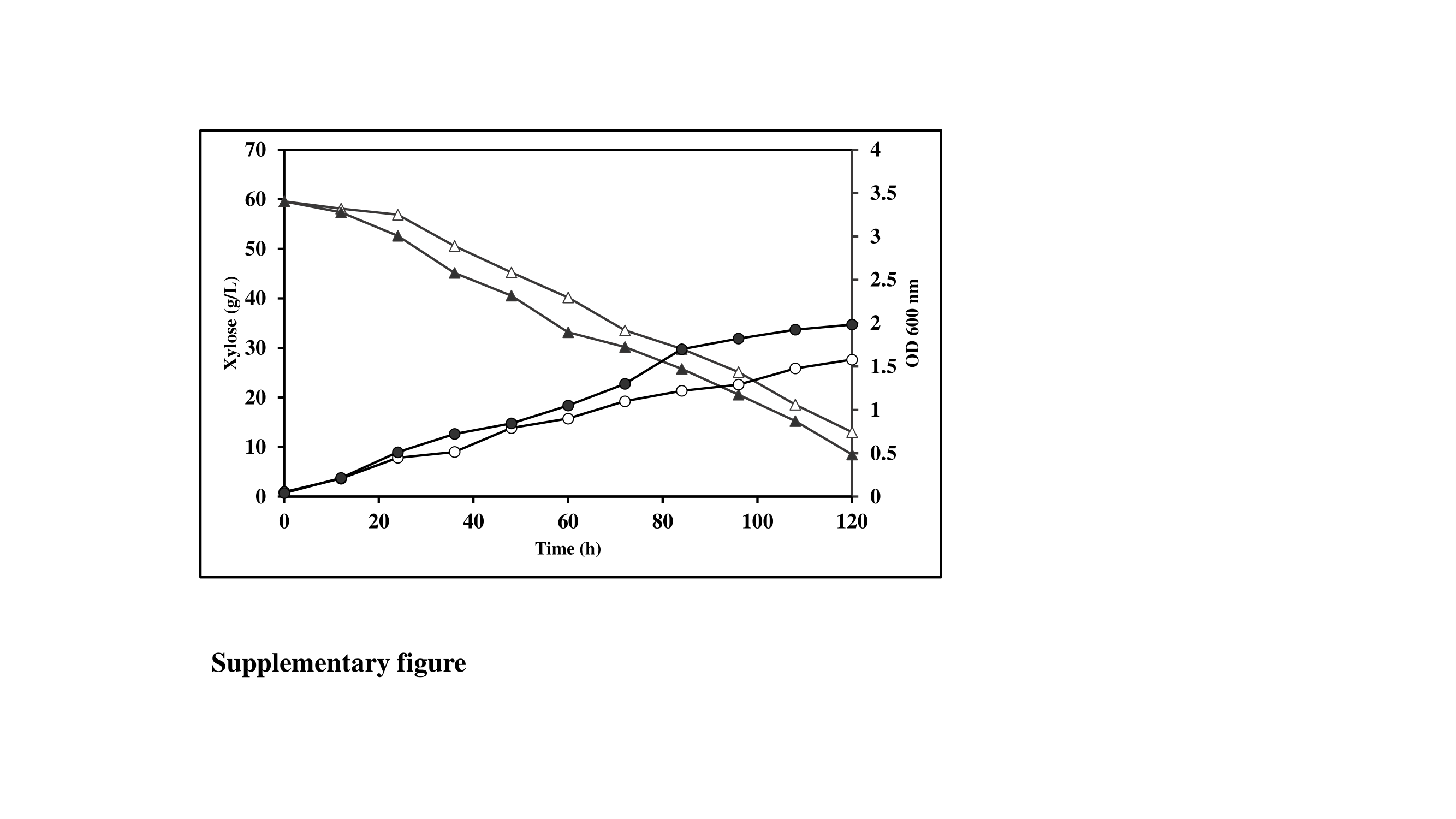

Supplement: Supplementary file 1 — Additional file 1: Figure S1. Growth (circles) and xylose consumption (triangles) by C. glutamicum ATCC 13032 (pVWEx1-xylB) (open symbols) and C. glutamicum ATCC 31831 (pVWEx1-xylB) (closed symbols) in CGXII medium containing 60 g/L xylose. [file 13568_2020_1003_MOESM1_ESM.tiff]
